# Megahertz non-contact luminescence decay time cryothermometry by means of ultrafast PbI_2_ scintillator

**DOI:** 10.1038/s41598-019-41768-z

**Published:** 2019-03-27

**Authors:** V. B. Mykhaylyk, H. Kraus, L. Bobb, R. Gamernyk, K. Koronski

**Affiliations:** 1 Diamond Light Source, Harwell Campus, Didcot, OX11 0DE UK; 2 University of Oxford, Department of Physics, Denys Wilkinson Building, Keble Road, Oxford, OX1 3RH UK; 3 Lviv National University, Physics Department, 8 Kyrylo and Mefodiy str, Lviv, 29005 Ukraine; 4 Institute of Physics, Polish Academy of Sciences A., Lotnikow 32/46, Warsaw, 02-668 Poland

## Abstract

Realtime *in situ* temperature monitoring in difficult experimental conditions or inaccessible environments is critical for many applications. Non-contact luminescence decay time thermometry is often the method of choice for such applications due to a favorable combination of sensitivity, accuracy and robustness. In this work, we demonstrate the feasibility of an ultrafast PbI_2_ scintillator for temperature determination, using the time structure of X-ray radiation, produced by a synchrotron. The decay kinetics of the scintillations was measured over the 8–107 K temperature range using monochromatic pulsed X-ray excitation. It is found that lead iodide exhibits a very fast and intense scintillation response due to excitons and donor-acceptor pairs, with the fast decay component varying between 0.08 and 0.5 ns – a feature that can be readily exploited for temperature monitoring. The observed temperature dependence of the decay time is discussed in terms of two possible mechanisms of thermal quenching – transition over activation barrier and phonon-assisted escape. It is concluded that the latter provides a better fit to the experimental results and is consistent with the model of luminescence processes in PbI_2_. We evaluated the sensitivity and estimated the accuracy of the temperature determination as ca. ±6 K at 107 K, improving to ±1.4 K at 8 K. The results of this study prove the feasibility of temperature monitoring, using ultrafast scintillation of PbI_2_ excited by X-ray pulses from a synchrotron, thus enabling non-contact *in-situ* cryothermometry with megahertz sampling rate.

## Introduction

Temperature is a fundamental property of matter and plays an important role in many physical, chemical and biological processes. Solutions to challenging problems that modern science tends to face often necessitate experimenting in harsh and/or hardly accessible environments where accurate and reliable temperature monitoring is difficult. As an example, temperature is a very important parameter when aiming to protect biological samples from radiation damage during experiments that utilise powerful ionising radiation produced by modern synchrotron light sources^1–4^, There is growing indication that intense irradiation applied to samples of microscopic size can significantly increase their temperature, which in turn causes sample degradation. This finding is likely to have even higher impact owing to the even higher brilliance that will result from planned upgrades of synchrotrons^5,6^,

A novel method for non-contact monitoring of temperature in a vacuum environment has recently been developed and deployed for experimenting at the long-wavelength macromolecular crystallography (MX) beamline I23 at the Diamond Light Source (DLS)^7^ The temperature is derived from changes in the luminescence decay characteristics of a Bi_4_Ge_3_O_12_ (BGO) scintillation sensor. Results from extensive testing have demonstrated the reliability and accuracy as well as the advantages of the technique^8^. In the temperature range of beamline operation (30–150 K) the error of temperature determination using a BGO scintillator is ±1.6 K. The absence of electrical connections makes this system fully compatible with both the vacuum environment and the necessity of swift replacement and manipulation of samples.

The method as it stands now has one intrinsic limitation, though. The luminescence decay time of the scintillation sensor used is within the 10^−6^–10^−3^ s range. This is a typical decay time for the majority of materials exhibiting thermally activated changes of luminescence properties at cryogenic temperatures. For measurements of the luminescence kinetics the technique employs a pulsed UV excitation source with a repetition rate of <1 kHz^8,9^, However, throughout experimenting with X-rays, high-energy ionising radiation excites spurious luminescence in the material of the sensor, which exceeds the useful signal by a few orders of magnitude and prevents determination of the luminescence decay, thus making the measurements unreliable. This limitation significantly affects the merit of the technique and precludes application of this method for *in-situ* experiments that require X-rays. Thus, the most interesting and important information about temperature changes induced by ionised radiation remains elusive.

It has been realised that the time structure of the X-rays produced by synchrotrons offers an elegant way to circumvent this limitation. During normal operation of the DLS, electron bunches from the synchrotron ring emit a series of sharp X-ray pulses (FWHM of Δt = 45 ps) at an interval of 2 ns. This gap can be used for measurements of very fast decay processes. Consequently, implementation of this idea requires a scintillator with ultrafast scintillation decay that also exhibits a significant change to that time constant in the temperature range of interest. The availability of a suitable scintillation material is key for this application. Motivated by this idea we initiated a search for a scintillator that satisfies these requirements.

Ultrafast temperature-dependent scintillations are observed in several semiconductors, e.g. CuI, HgI_2_, PbI_2_, ZnO, CdS, etc^10^. Lead iodide has been investigated as a scintillator throughout the past century and there is a wealth of published data evidencing that it is very promising for application at cryogenic temperatures^11–14^, Thus, we anticipated that due to a very high light yield of 40000 ph/MeV at 14 K and a prominent variation of the decay time constant over the temperature range of interest, 10–100 K^14^, PbI_2_ can offer a viable solution for the application under consideration. In the work presented here we report on the results of a feasibility study and characterisation of a PbI_2_ scintillation sensor for non-contact luminescence cryothermometry using pulsed X-ray excitation of a synchrotron light source.

## Results

Lead iodide is crystallised in a rhombohedral crystal structure (space group P-3m1) with lattice constants a = b = 4.56 Å, c = 6.98 Å, α = β = 90° and γ = 120°. The crystal structure is formed by edge-sharing layers of PbI_6_ octahedrons, running parallel to the *ab*-plane. The neighbouring layers are linked by Van der Waals forces, and that explains the high susceptibility of PbI_2_ to cleaving. It is a narrow-band semiconductor with a direct band gap of 2.53 eV, density 6.16 g/cm^3^ and melting point of 402 °C.

There are several prior studies of luminescence and scintillation properties of PbI_2_ stating that at cryogenic temperatures the emission arises from recombination of excitons and donor-acceptor pairs^11,15–19^. When excited by X-rays, PbI_2_ exhibits near-edge narrowband emission peaking at 520 nm with a very pronounced temperature dependence^13,14^. Time-resolved studies carried out at 5 K showed that the radiative relaxation of excitons in PbI_2_ exhibits a non-exponential decay with sub-nanosecond decay time constant. The latter is caused by the polariton effect i.e. a process controlled by excitons-phonon interaction^20^. The decay time constant measured at excitation with X-rays are typically different from those observed at optical excitation. This difference is readily explained by the fact that X-rays produce ionisation tracks with a high density of charged particles, which transfer excitation energy to donor-acceptor pairs.

**Figure 1:**
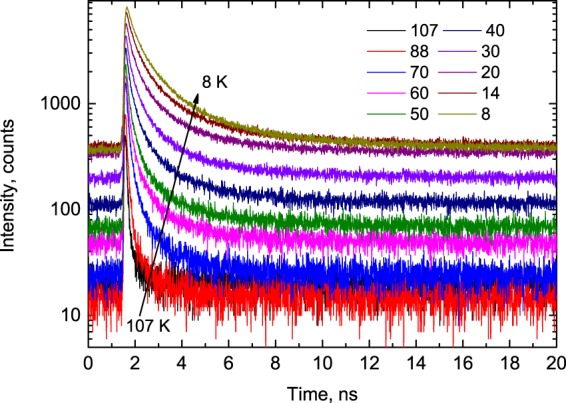
Decay curves of X-ray luminescence measured in the PbI_2_ crystal at different temperatures from 8 to 107 K. The luminescence is excited by 60 ps pulses of synchrotron radiation (E = 14 keV).

A key feature of the sensor for its use in non-contact thermometry is an ultrafast luminescence decay time constant. Therefore, we begin with presenting results on the X-ray-excited luminescence decay as a  function of temperature. Figure 1 displays the change of the decay time response of PbI_2_ emission to pulsed X-ray excitation as the temperature decreases from 107 to 8 K.

**Figure 2:**
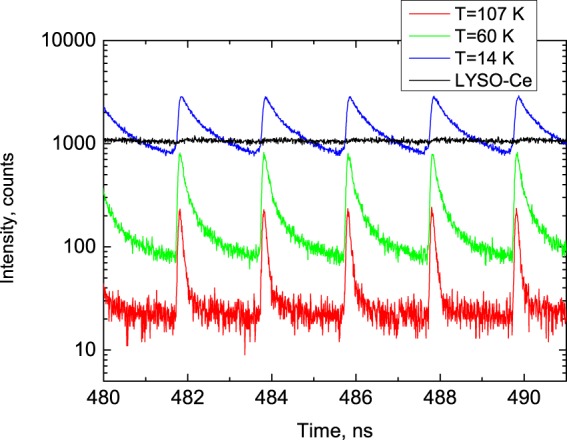
The sequence of X-ray pulses, emitted by the electron bunches of the synchrotron ring (time interval 2 ns, FWHM = 45 ps) as detected using a PbI_2_ at T = 14 K (blue), 60 K (green), 107 K, (red) and LYSO-Ce scintillator (T = 292 K, black). The graphs demonstrate the exceptionally good timing resolution of the scintillation response for PbI_2_ crystal as opposed to the completely saturated and featureless signal from LYSO-Ce.

The decay curves exhibit very fast, non-exponential kinetics which is indicative of a complex emission process involving the radiative annihilation of free and bound excitons in semiconductors^14,21–24^. Inspection of Fig. 1 proves that the decay curves of PbI_2_ exhibit prominent change with temperature. Most obvious is the dramatic rise of the amplitude of the scintillation pulse. As the temperature decreases from 107 to 8 K there is an almost ten-fold increase of the peak intensity, which is consistent with previous observations^13,14^. Careful inspection of the data reveals that the shape of the scintillation pulse in PbI_2_ also undergoes noticeable changes with cooling, indicating a slowing down in the recombination dynamics. This is due to the reduction of the luminescence decay rate with a decrease of temperature, commonly observed in many scintillation materials. Another feature of PbI_2_, which is crucial for the envisaged application, is the ultrafast decay time that allows measurements of decay curves over a narrow time interval between consecutive X-ray pulses. Figure 2 displays the sequence of X-ray pulses from the synchrotron (FWHM = 45 ps, interval Δt = 2 ns) detected by PbI_2_ at different temperatures. This is a very clear demonstration of the possibility for such measurements with a megahertz sampling rate.

**Figure 3:**
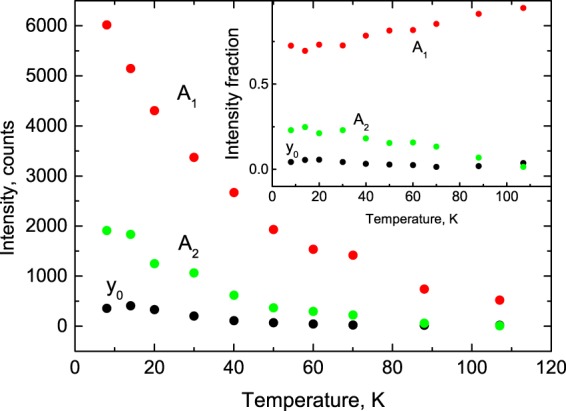
Temperature dependence of amplitudes obtained from the fitting of the decay curves of PbI_2_ with a sum of two exponential functions: $y={A}_{1}\exp (-t/{\tau }_{1})+{A}_{2}\exp (-t/{\tau }_{2})+{y}_{0}$. The inset shows fractional contributions of the components to the total emission amplitude.

**Figure 4:**
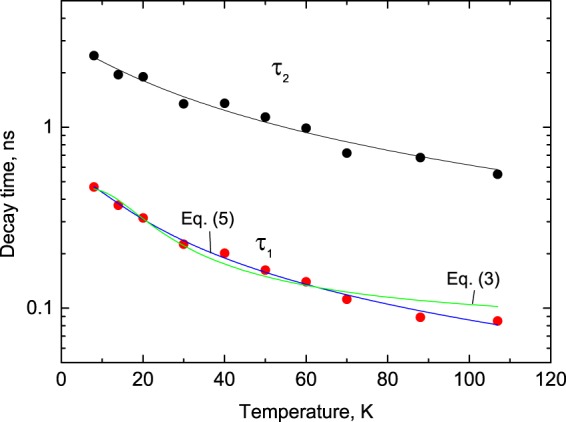
Temperature dependence of decay time constants of PbI_2_. The lines show the best fit of the temperature dependence to different models a) the model of thermal activation over an energy barrier (Equation (3), green line) and b) multi-phonon scattering (Equation (5) blue line). The best fit for τ_1_=ƒ(T) was obtained using Equation (5) with the following parameters:  *τ*_r_=3 × 10^6^ s, ${\Gamma }_{0}$ = 1.90 ± 0.08 × 10^9^ s^−1^, *E*_*ph*_ = 1.54 meV, *n* = 1.

Given that non-contact thermometry relies totally on the changes of the decay curves with temperature, all these features are very important from the viewpoint of the present study. Therefore, for a more quantitative evaluation of these properties and trends, we fitted the measured decay curves with a sum of exponential functions: $f(t)={\Sigma }_{i}{A}_{i}\exp (-t/{\tau }_{i})+{y}_{o}$, where *A*_*i*_ is the amplitude, *τ*_*i*_ the decay time constant and *y*_0_ the background. It should be noted that the multi-exponential fit is widely applied to characterise the processes of radiative decay of excitons and donor-acceptor pairs in semiconductors^13,21–25^. The quality of the fit was only marginally different between two- and three exponential fits. Two exponentials and a constant background is sufficient for an adequate representation of the measured decay curves. The fitting parameters for PbI_2_ as functions of temperature are shown in Figs 3 and 4.

Analysis of these plots reveals further details of the temperature evolution of the luminescence kinetics of the crystal. The total amplitude of the fast (*A*_1_), and slow (*A*_2_) emission components, as well as the background (*y*_0_) exhibit a pronounced increase with cooling (see Fig. 3). Interestingly, for the factional contributions of the two emission components, the opposite trends are observed (see inset to Fig. 3). With cooling, the fractional contribution of the slow component in the decay curve becomes more pronounced, rising from 0 to 25%, while the background increases from 0 to 5%, and the fractional contributions of the fast component gradually reduce from 100 to 70%. Figure 4 shows the temperature dependence of the fast (*τ*_*i*_) and slow (*τ*_2_) decay constants. As expected, both decay constants increase with the crystal cooling over the entire measured range (8–107 K). It is this characteristic of PbI_2_ emission that is of primary importance for the non-contact temperature monitoring.

## Discussion

The data in Fig. 4 demonstrate a relatively large dependence of the decay time constant on temperature due to considerable thermal quenching. The origin of the observed dependence of the decay time versus temperature can be understood by considering the dynamics of radiative and non-radiative transitions between the excited and ground states of the emission center. In the framework of this consideration, the measured transition rate (inverse of luminescence decay constant *τ*) can be determined as sum of radiative (*k*_*r*_) and non-radiative (*k*_*nr*_) rates:

1 $$\frac{1}{\tau }={k}_{r}+{k}_{nr}=\frac{1}{{\tau }_{r}}+\frac{1}{{\tau }_{nr}}$$

In insulators where luminescence originates from radiative decay of emission centers (impurity or defect) the changes of decay time with temperature are traditionally attributed to the process of depopulation of excited levels of the emission center due to thermally induced transition of excited particles over the energy barrier that leads to the non-radiative decay. The rate associated with the non-radiative process exhibits a strong temperature dependence, thus controlling the variation of the non-radiative decay with temperature:

2 $$\frac{1}{{\tau }_{nr}}=Kexp(\frac{-\Delta E}{kT})$$

where *K* is the probability of non-radiative decay, Δ*E* is the activation energy for the non-radiative transitions, and *k* is Boltzmann’s constant. Substituting (2) into (1) brings about the classical formula which allows to describe many practical cases of the τ = ƒ(T) dependence:^9^

3 $$\frac{1}{\tau }=\frac{1}{{\tau }_{r}}+Kexp(\frac{-\Delta E}{kT})$$

A complementary mechanism of thermal de-activation due to multi-phonon scattering is often considered in semiconductors, where the luminescence originates from recombination of free carriers with donors and/or acceptors^26–28^. From the viewpoint of the related model, the non-radiative recombination occurs because of phonon-assisted escape of carriers out of the emission centre. This is possible because multiple types of donor and acceptor centers create in the bandgap of semiconductor a multitude of intermediate levels that can be involved in phonon-assisted processes. There, the temperature dependent change of the non-radiative decay rate is defined as thermally aided scattering of carriers via phonon emission. The theoretical expression for the non-radiative decay is given by the following expression:^29^

4 $$\frac{1}{{\tau }_{nr}}={\Gamma }_{0}{(\frac{1}{exp(\frac{{E}_{ph}}{kT})-1}+1)}^{n},$$

where ${\Gamma }_{0}$ is the probability of non-radiative decay at T = 0, *E*_*ph*_ is the phonon energy and *n* is the number of phonons involved in the process. After rearranging (4) and substituting into (1) the expression for the temperature dependence of the luminescence decay constant it becomes

5 $$\frac{1}{\tau }=\frac{1}{{\tau }_{r}}+{\Gamma }_{0}{(1-exp(\frac{-{E}_{ph}}{kT}))}^{-n}.$$

We tested both models to fit our experimental data (see Fig. 4) and found that Equation (5) gives a superior result: the goodness of fit defined as chi square is 0.978 for the first model and 0.992 for the multi-phonon scattering model. This finding provides convincing evidence that phonon-assisted processes control thermally induced effects occurring in PbI_2_ at low temperatures.

Having established the analytical expression for the τ = ƒ(T) dependence in PbI_2_ we can now evaluate the potential of the material for non-contact measurements of temperature under the experimental conditions that provided the results under discussion. The most important characteristics of performance of a temperature sensor are sensitivity and accuracy. The sensitivity is determined as a derivative Δτ/ΔT that determines the slope of the τ = ƒ(T) curve. It is desirable to have a sufficiently large value of this parameter over the entire temperature range of interest or in other words a sharp fall-off of the decay time with temperature. From the data in Fig. 4 it is fairly obvious that the fast decay constant exhibits the steeper slope. Moreover, the error of fitting the fast decay constant is almost a factor two smaller over the entire temperature range of interest in comparison with the slow decay constant. This is because the amplitude of the fast component is much higher, resulting in a higher signal-to-noise ratio. Therefore, the fast decay constant is invariably preferred for temperature monitoring.

**Figure 5:**
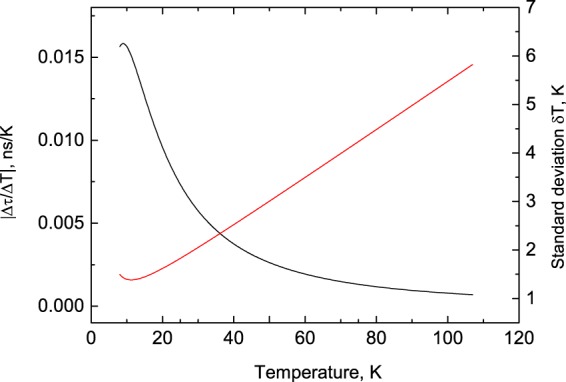
The temperature variation of the sensitivity |(Δτ/ΔT)| (black) and uncertainty of temperature measurements (red) using a PbI_2_ scintillation sensor. The curves are calculated using the fitted τ_1_ = ƒ(T) dependence, displayed in Fig. 4.

Using the results of the fit to the multi-phonon model, we calculated the variation of Δτ/ΔT with temperature. According to the plot displayed in Fig. 5, the sensitivity increases from 6 × 10^−4^ to 1.5 × 10^−2^ ns/K as temperature decreases from 107 to 8 K. It is pertinent to remark that the  sensitivity also has a direct impact on the precision of temperature measurements. To estimate the accuracy of the temperature determination using PbI_2_ we analyzed multiple traces of decay curves similar to the one displayed in Fig. 2 and estimated that the error in the measurement of the fast decay time constant is *ε* = ±6%. This allows the calculation of the uncertainty of determining the temperature *δT* from the τ = ƒ(T) dependence and sensitivity curve Δτ/ΔT = ƒ(T) using the following formula^30^:

6 $$\delta T=\varepsilon \tau (T){(\frac{\Delta \tau }{\Delta T})}^{-1}.$$

Figure 5 shows that the measurement errors change significantly over the examined temperature range. The uncertainty of the temperature determination is ±1.4 K at 8 K but gradually increases to ±6 K at 107 K. The enhanced magnitude of the error towards higher temperatures is expected; it is due to the reduced sensitivity of PbI_2_ in this range. At this stage the obtained accuracy in temperature reading is worse than what is typically achieved by non-contact luminescence decay techniques that utilise conventional near room temperature sensors (±1 K or better)^31–34^. Hovewer, it is comparable with the accuracy demonstrated by the method when used for the measurements of cryogenic temperatures^35,36^. It is also expected that the  accuracy can be significantly improved through the optimisation of hardware, detection method and data analysis. Therefore, we are confident that the PbI_2_ scintillator with subnanosecond time resolution can open a new domain for application in tempereature measurements, enabling fast non-contact luminescence decay time thermometry.

## Conclusion

In this work we investigated the temperature-induced changes in the kinetics of ultrafast scintillations in PbI_2_ excited by pulsed monochromatic 14 keV X-rays from a synchrotron source. When cooled to below 100 K this material exhibits intense emission with very fast decay at X-ray excitation. We monitored changes of the luminescence kinetics with cooling from 107 to 8 K and determined the temperature dependence of the decay characteristics by fitting the measured decay curves by a sum of two exponential functions. It has been found that between 107 and 8 K, lead iodide exhibits a very prompt and intense scintillation response, with the fast decay component varying between 0.08 and 0.5 ns, making this scintillator suitable for temperature monitoring.

To explain the observed temperature change of the decay time we examined two theoretical models. By fitting the measured τ = ƒ(T) dependence to the pertinent analytical equations given by the models, we demonstrated that in PbI_2_ temperature changes of the luminescence decay time are governed by the non-radiative recombination process due to phonon-assisted escape of carriers out of the emission centre.

Motivated by the idea of non-contact decay time thermometry we evaluated the sensitivity of the PbI_2_ sensor and estimated the accuracy. It is found that the error of temperature determination is ca. ±6 K at 107 K reducing to ±1.4 K at 8 K. Overall this clearly demonstrates the potential of the PbI_2_ scintillator for temperature monitoring by measuring the luminescence decay curve excited by X-ray pulses. Crucially, the ultrafast emission decay time of PbI_2_ allows measurements to be done using the standard bunch structure of X-ray emission produced by DLS and other synchrotrons. This proves the feasibility of the fast non-contact luminescence decay time cryothermometry with megaherthz sampling rate and we aim to develop this technique further.

## Materials and Methods

The sample of PbI_2_ with approximate dimensions 4 × 5 × 1.5 mm^3^ was cleaved from an ingot that has been grown by using the Bridgeman technique in a quartz ampule. The scintillation decay curves of the crystals were measured at the beamline B16 of DLS, using a 14 keV monochromatic X-ray beam from the synchrotron. The measurements were carried out at a special beam condition suitable for time-resolved experiments^37^ by triggering on a single X-ray pulse with FWHM of Δt = 60 ps, separated from the following pulse by a 250 ns gap. The sample was glued with silver adhesive to the holder of a continuous-flow, He-cryostat (Oxford Instruments). A controller stabilized the temperature in the cryostat using a PID feedback loop with a Si-diode as a temperature sensor and a resistive heater. The cryostat was attached to an XYZ-translation stage to facilitate swift alignment. The cryostat has an 0.2 mm thick aluminised Mylar window, transparent for 14 keV X-rays. Through this window the X-ray beam 1 × 1 mm^2^ with a flux of ~10^9^ ph/(s·mm^2^) was irradiating the sample placed at 45° to the incoming radiation while the luminescence was collected in reflection mode at 45° through a quartz window. The emission was detected using an ID100 single photon counting detector sensitive over a 400–900 nm spectral range and a Picoharp 300 time-correlated single photon counting module.

## References

[1] Ponomarenko O (2011). Radiation-induced melting in coherent X-ray diffractive imaging at the nanoscale. Journal of Synchrotron Radiation.

[2] Garman EF, Weik M (2017). X-ray radiation damage to biological macromolecules: further insights. Journal of Synchrotron Radiation.

[3] Russi S (2017). Conformational variation of proteins at room temperature is not dominated by radiation damage. Journal of Synchrotron Radiation.

[4] Warkentin MA (2017). Lifetimes and spatio-temporal response of protein crystals in intense X-ray microbeams. IUCrJ.

[5] Pacchioni, G. An upgrade to a bright future, *Nature Reviews Physics*, pp. 1–2 (2019).

[6] Eriksson M, Van Der Veen JF, Quitmann C (2014). Diffraction-limited storage rings-A window to the science of tomorrow. Journal of Synchrotron Radiation.

[7] Wagner A, Duman R, Henderson K, Mykhaylyk V (2016). In-vacuum long-wavelength macromolecular crystallography. Acta Crystallography D.

[8] Mykhaylyk VB, Wagner A, Kraus H (2017). Non-contact luminescence lifetime cryothermometry for macromolecular crystallography. Journal of Synchrotron Radiation.

[9] Ahmed N (2018). Characterisation of tungstate and molybdate crystals ABO_4_ (A = Ca, Sr, Zn, Cd; B = W, Mo) for luminescence lifetime cryothermometry. Materialia.

[10] Derenzo SE, Bourret-Courshesne E, Bizarri G, Canning A (2016). Bright and ultra-fast scintillation from a semiconductor?. Nuclear Instruments and Methods in Physics Researches A.

[11] Voloshinovskii, A. S., Pidzyrailo, M. S., Mikhailik, V. B. & Rodnyi, P. A. Luminescence properties of PbX_2_ (X = F, Cl, Br, I) crystals., In Proceedings of Material Research Society Spring Meeting, Scintillators and Phosphor Materials Symposium, San Francisco, **348**, 149–154 (1994) .

[12] Moses WW (2004). Observation of fast scintillation of cryogenic PbI2 with VLPCs. IEEE Transactions on Nuclear Science.

[13] Derenzo SE, Weber MJ, Klintenberg MK (2002). Temperature dependance of the fast, near-band-edge scintillation from CuI, HgI2, PbI2, ZnO:Ga and CdS:In. Nuclear Instrumnts and Methods in Physics Researches A.

[14] Derenzo SE (2013). Experimental and theoretical studies of donor–acceptor scintillation from PbI2. Journal of Luminescence.

[15] Kleim R, Raga F (1969). Exciton luminescence in lead iodide lifetime, intensity and spectral position dependence on temperature. Journal of Physics and Chemistry of Solids.

[16] Lévy F, Mercier A, Voitchovsky J-P (1974). Band-edge photoluminescence of PbI2. Solid State Communications.

[17] Bibik VA, Davydova NA (1991). Donor‐acceptor emission in PbI2 crystals. Physica Status Solidi A.

[18] Novosad SS, Novosad IS, Matviishin IM (2002). Luminescence and Photosensitivity of PbI2 Crystals. Inorganic Materials.

[19] Furyer MS (2010). Study of the photoluminescence and photoelectric properties of Pb1−xCdxI2 alloys. Journal of Applied Physics.

[20] Makino T, Watanabe M, Hayashi T, Ashida M (1998). Time-resolved luminescence of exciton polaritons in PbI2. Physical Review B.

[21] Wang W, Lin AS, Phillips JD, Metzger W (2009). Generation and recombination rates at ZnTe:O intermediate band states. Applied Physics Letters.

[22] Wakita K, Nishi K, Ohta Y, Nakayama N (2002). Time-resolved photoluminescence studies of free excitons in CuInS2 crystals. Applied Physics Letters.

[23] Murphy GP, Zhang X, Bradley AL (2016). Temperature-dependent luminescent decay properties of CdTe quantum dot monolayers: impact of concentration on carrier trapping. Journal of Physical Chemistry C.

[24] Mykhaylyk, V. B., Kraus, H. & Saliba, M. Bright and fast scintillation of MAPbBr_3_ at low temperatures. *Materials Horizons*, 10.1039/c9mh00281b (2019).

[25] Zhang YX (2017). Photoluminescence quenching of inorganic cesium lead halides perovskite quantum dots (CsPbX3) by electron/hole acceptor. Physical Chemistry Chemical Physics.

[26] Ruan XL, Kaviany M (2005). Enhanced nonradiative relaxation and photoluminescence quenching in random, doped nanocrystalline powders. Journal of Applied Physics.

[27] De Giorgi M (2001). Capture and thermal re-emission of carriers in long-wavelength InGaAs/GaAs quantum dots. Applied Physics Letters.

[28] Man MT, Lee HS (2015). Discrete states and carrier-phonon scattering in quantum dot population dynamics. Scientific Reports.

[29] Risenberg LA, Moss HW (1968). Multiphoton orbit lattice relaxation of excited states of rare-earth ions in crystals. Physical Review.

[30] Chambers MD, Clarke DR (2009). Doped oxides for high temperature luminescence and lifetime thermometry. Annual Review of Materials Research.

[31] Allison SA, Gillies GT (1997). Remote thermometry with thermographic phosphors: Instrumentation and application. Review of Scientific Instruments.

[32] Homeyer KE (2015). Diamond contact-less micrometric temperature sensors. Applied Physics Letters.

[33] Brübach J, Pflitsch C, Dreizler A, Atakan B (2013). On surface temperature measurements with thermographic phosphors: A review. Progress in Energy and Combustion Science.

[34] Yakunin, S. *et al*. High-resolution remote thermography using luminescent low-dimensional tin halide perovskites (in print). Nature Methods (2019). 10.1038/s41563-019-0416-2 31263225

[35] Mahata MK (2016). Demonstration of temperature dependent energy migration in dual-mode YVO4: Ho3+/Yb3+ nanocrystals for low temperature thermometry. Scientific Reports.

[36] Cai T, Kim D, Kim M, Liu YZ, Kim KC (2017). Two-dimensional thermographic phosphor thermometry in a cryogenic environment. Measuremnts Science and Technology.

[37] Rutherford ME (2016). Evaluating scintillator performance in time-resolved hard X-ray studies at synchrotron light sources. Journal of Synchrotron Radiation.

